# Naïve, uninformed and sexually abused: circumstances surrounding adolescent pregnancies in Malawi

**DOI:** 10.1186/s12978-023-01655-3

**Published:** 2023-08-06

**Authors:** Elita Chamdimba, Caroline W. Kabiru, Boniface Ayanbekongshie Ushie, Alister Munthali, Chrissie Thakwalakwa, Anthony Idowu Ajayi

**Affiliations:** 1 Center for Social Research, University of Malawi, P.O. Box 280, Zomba, Malawi; 2 Sexual, Reproductive, Maternal, New-Born, Child and Adolescent Health (SRMNCAH) Unit, African Population and Health Research Center, Manga Close, Nairobi, Kenya

**Keywords:** Unintended pregnancy, Adolescent pregnancy, Sexual violence, Contraceptives, Malawi, Parenting girls, Parenting boys

## Abstract

**Background:**

Pregnancy and childbearing in adolescence could negatively affect girls’ health and socio-economic wellbeing across the life course. Previous studies on drivers of adolescent pregnancy in Africa have not fully considered the perspectives of parents/guardians vis-à-vis pregnant and parenting adolescents. Our study addresses this gap by examining pregnant and parenting adolescents’ and parents/guardians’ narratives about factors associated with early and unintended pregnancy.

**Methodology:**

The descriptive study draws on qualitative data collected as part of a larger mixed-methods cross-sectional survey on the lived experiences of pregnant and parenting adolescents. Data were collected between March and May 2021 in Blantyre, Malawi, using semi-structured interview guides. We interviewed 18 pregnant and parenting adolescent girls, 10 parenting adolescent boys, and 16 parents/guardians of pregnant and parenting adolescents. Recorded interviews were transcribed verbatim into the English language by bilingual transcribers. We used the inductive-thematic analytical approach to summarize the data.

**Findings:**

The data revealed several interconnected and structural reasons for adolescents’ vulnerability to early and unintended pregnancy. These include adolescents’ limited knowledge and access to contraceptives, poverty, sexual violence, school dropout, COVID-19 school closures, and being young and naively engaging in unprotected sex. While some parents agreed that poverty and school dropout or COVID-19 related school closure could lead to early pregnancies, most considered stubbornness, failure to adhere to abstinence advice and peer influence as responsible for adolescent pregnancies.

**Conclusion:**

Our findings contribute to the evidence on the continued vulnerability of girls to unintended pregnancy. It highlights how parents and adolescents hold different views on reasons for early and unintended pregnancy, and documents how divergent views between girls and their parents may contribute to the lack of progress in reducing adolescent childbearing. Based on these findings, preventing unintended pregnancies will require altering community attitudes about young people’s use of contraceptives and engaging parents, education sector, civil society organizations and community and religious leaders to develop comprehensive sexuality education programs to empower in- and out-of school adolescents.

## Introduction

Adolescent pregnancy is a major public health problem worldwide, with 42.5 births per 1,000 girls aged 15–19 years in 2021 [1]. However, adolescent pregnancies are highest in low and middle-income countries, with an estimated 12 million births among girls 15 to 19 years and 777,000 among girls younger than 15 years annually. About 10 million of these births result from unintended pregnancies. The highest adolescent pregnancy rates are in East, West and Central, and Southern Africa, with 98 births per 1000 girls [2]. Malawi is among the leading countries globally in adolescent childbearing, with a rate of 138 births per 1000 girls [3]

Early and unintended pregnancy has negative health effects despite adolescence being characterized as the healthiest period in an individual's life. Complications arising from pregnancy and childbirth are the leading cause of death for 15–19-year-old girls globally [4]. The risk of dying from pregnancy-related causes is twice for adolescents compared to adult women [5, 6]. Early and unintended pregnancy also has socio-economic consequences. For instance, pregnant adolescents are more likely to drop out of school without prospects of reentry after delivery [7]. The negative consequences of early and unintended pregnancy are more severe for girls in the poorest households [8].

Only a few studies have explored, from the perspective of pregnant and parenting girls, why adolescents are vulnerable to early and unintended pregnancy [9]. For example, a mixed-methods study by Ajayi et al., [9] showed that higher proportions of unintended adolescent pregnancies in Kenya were among girls out-of school, married and not using contraceptives. Other studies identified early sex and marriage, wealth status, low contraceptive use, low educational levels, low socio-economic status, limited knowledge of sexual and reproductive health, and physical/sexual violence as drivers of adolescent pregnancy [10–12]. Interestingly, studies show that adolescent pregnancy and school dropout usually have the same drivers, including early sexual debut and poverty. In addition, a two-way relationship exists in the sense that a girl dropping out of school increases the likelihood of adolescent pregnancy and vice versa [13].

While several studies [11, 14, 15] have been conducted on adolescent pregnancy in Malawi to understand why adolescent girls are vulnerable to early and unintended pregnancy, little is known about the drivers of adolescent pregnancy from the voices and perspectives of those who are pregnant and parenting as well as their parents. This paper, therefore, closes a gap in the literature by exploring the reasons for the vulnerability of adolescents to early and unintended pregnancy from a qualitative perspective through the voices of pregnant and parenting adolescents. We focus on pregnant and parenting adolescents' narratives to understand in-depth why they got pregnant. In addition, we juxtaposed the reasons adduced by pregnant and parenting adolescents with those of their parents/guardians to illustrate disjunction and differences in their views.

## Theoretical underpinning

The theoretical perspective underpinning this study is the socio-ecological model (SEM), which acknowledges individual, interpersonal (family/peers), structural factors influencing young people’s choices [16]. The individual-level characteristics that could potentially be associated with early and intended pregnancies include age, gender, knowledge and attitudes about contraceptives, and negotiation skills about sexual consent. The next layer of the SEM covers the interpersonal relationship level, which for adolescents encompasses their support and social networks such as parents/guardians, partners, friends, peers, and teachers. The SEM has other exosystem and macrosystem levels (community, institutions, and societal levels), however, the findings presented in this paper are largely focused on the microsystem and mesosystem of individual and interpersonal levels.

## Methodology

### Study site

We conducted the study in Blantyre, a district in southern Malawi. Between 2019 to 2020 the southern region districts of Malawi experienced an increase in child marriage, with Blantyre reporting the highest at 97.4% [17]. As of 2020, the number of newly reported child marriages in Blantyre was 309 from only eight in 2019 [17]. Given that the interrelationship between drivers of adolescent pregnancy and child marriage [18], Blantyre district was selected as the study site. In terms of the socioeconomy of Blantyre, poorer households make up 64% of the district's population since their international wealth index (IWI) value is under 50 while, the poorest households make up 43.5% of Blantyre with an IWI value below 35 [19].

### Study design

Most research on adolescent pregnancies adopted a quantitative design [10]. Our study aims to contribute to the literature a more in-depth understanding of the circumstances surrounding adolescent pregnancies in Malawi as captured by the experiences and perceptions of girls, boys, and their parents/guardians. Therefore, the most suitable research philosophy for this study is an interpretivism paradigm [20]. As a result, we adopted a descriptive qualitative design involving semi-structured interviews with pregnant and parenting adolescents and parents/guardians.

### Positionality

We, the authors of this paper, are Global South researchers based in southern and east Africa, some specifically from Malawi. As a team and individually, we have years of experience in qualitative research with adolescents at risk such as adolescent mothers, school dropouts, and girls in child marriage among other groups. Our insider positionality comes from our identity as sub-Saharan African researchers. Hence any pre-understanding of the topic of study is derived from the findings in our previous research [9, 18, 21]. Our familiarity with the context as well as the issues of focus [22] added value to the research by facilitating data collection and interpretation of the findings.

### Participant selection

Non-probability sampling was used to select participants. Given that this study was seeking diverse views and since saturation can be gained with small sample size [23], purposive sampling was applied to select participants from various contexts including rural and urban settings and different ages. The inclusion criteria were anyone aged 10–19 years who was either pregnant, had ever been pregnant, or had a biological child. Those who were not mentally capable to hold a conversation at the time of the interview were excluded. Participants who met the predefined selection criteria were identified in the communities, approached face-to-face, and requested to participate in the study. Eighteen pregnant and parenting adolescent girls, 10 parenting boys, and 16 parents/guardians participated in the study.

### Participants’ demographic characteristics

Among the 18 pregnant and parenting adolescent girls, three became pregnant at 14 years, five at 16, three at 17, and the rest at 18. At the time of the interviews, five of the girls were aged 17 years and below, while 13 were aged 18–19 years. All but one of the girls described their pregnancies as unintended. Fourteen of the girls were from rural areas while four were from urban areas. At the time of the interview, most of the girls (10) were living with their parents while the rest were living with their partner (three), living alone (three), or living with a foster parent/guardian (two). In terms of marital status, one of the girls was married, while the rest were unmarried. One of the girls was still in school, while the rest of the girls were school dropouts.

The parenting boys in this study were aged 18–19 years. Seven of them resided in rural areas while three were from urban areas. One boy was married while the rest were unmarried.

The 16 parents/guardians that participated in this study were aged 35–45 years. Most resided in rural areas (12).

### Data collection

The interviews were conducted by 12 research assistants (five males and seven females). The research assistants had a minimum of a bachelor’s degree and experience facilitating semi-structured interviews in the local language, Chichewa. The strength of this team of research assistants is that all were Malawians and some specifically from Blantyre. All were fluent in Chichewa and some were young mothers. Their positionality facilitated the study in building rapport with participants and giving them a better grasp of the issues. They were trained specifically for this study and participated in pre-testing the interview guides before fieldwork. All interviews were audio-recorded with written informed consent from the participants. We conducted face-to-face interviews at the participants’ homes, either inside or outside the house, depending on which setting offered more privacy. Given that data collection occurred during the COVID-19 pandemic, our research team adhered to COVID-19 prevention measures by keeping a safe distance, wearing face masks, and frequently sanitizing during the interviews. The interviews took place in Chichewa, the local language, and interviews lasted 20–30 min on average.

The audio files were transcribed directly into English using bilingual transcribers. While the interviews covered a range of questions designed to elicit the lived experiences of pregnant and parenting adolescents and their parents’ views, the analysis in this study focused on circumstances surrounding adolescent pregnancies as narrated by adolescents and what their parents considered responsible for it. As such, we analyzed responses to one main question: *what do you consider to be the main reason for the pregnancy*?

### Ethics considerations

The University of Malawi Research Ethics Committee (UNIMAREC) approved the study protocol and materials. Before commencing data collection, we undertook community entry through the Blantyre District Commissioner’s office, Blantyre Police, Blantyre City Chief Executive Office, and the District Social Welfare Office. At the community level, we introduced the study to community leaders such as village headperson and sought their permission to conduct the study. Participation in this study was voluntary. Before administering the study tools, written informed consent was obtained from participants – specifically parents and guardians, adolescents aged 18 and above and married adolescents. Assent were obtained from unmarried minors and their parents provided written informed consent. Participants’ rights to privacy, confidentiality and anonymity were ensured through this study Confidentiality was ensured by de-identifying the data. Privacy was ensured by conducting the interviews in private places free from others listening in. A distress protocol was developed for this study and used in training research assistants to identify signs of possible distress among participants and refer them for counseling services.

### Data analysis

Transcripts were coded by three trained coders using NVivo12 software for pre-analysis. EC and AIA developed a coding tree to analyze the transcripts solely for this paper. First, EC and AIA read the transcripts for familiarization and a second time for immersion into the stories on circumstances surrounding adolescent pregnancies and who and what their parents believed were responsible. After full immersion into the data, we independently coded the data reflexively, using the inductive method of narrative analysis. EC and AIA derived the themes from the codes and met later to agree on the final themes.

## Findings

As reported in previous studies [24–26], a vast majority of adolescent pregnancies are unintended, occurring outside of formal union and when many were still in school. It is therefore important to consider this context in understanding reasons for adolescent vulnerability to early and unintended pregnancy. Overall, our analysis reveals a significant divergence between the views of pregnant and parenting adolescents and their parents on reasons for adolescent pregnancies. However, we observed that both adolescents and their parents agree on the contribution of poverty to early and intended pregnancies. We begin by presenting adolescents’ narratives on reasons for their pregnancies given their views differ significantly from their parents’. While circumstances surrounding each adolescent pregnancy tend to differ, common themes emerging from their narratives on reasons for their pregnancy include being young and naively in love, lack of contraceptives, sexual violence, poverty, school dropouts and extended periods out of school. These factors are interconnected and more frequently combine to exacerbate adolescents’ vulnerability to unintended pregnancy.

### Adolescents’ perceptions about the drivers of pregnancies

#### Being young and naïve

Most adolescents blamed their naivety for their unintended pregnancies. For much of the interviews, they would reply to questions about why they got pregnant by stating they were young and naïve. For example, recalling how she got pregnant, one participant stated: *“I was childish and too naïve at that time.” (Participant (4), Female, 19 years, Blantyre Rural). B*y stating they were young and naïve, they meant that they were inexperienced, impressionable, and impulsive. They naively took irrational risks they otherwise would not have taken. In other words, they naively engage in unprotected sex without carefully considering its consequences. Many adolescents narrated how they had sex carelessly without thinking, as recounted by a 19-year-old adolescent father residing in a rural area: “*the methods were there but at that time you know we were in love, so we never thought of using any contraceptives. And we continued making love like that without thinking, in the end, it just unexpectedly happened that she got pregnant. mmmm [yes]!” (Participant (1), Male, 18 years, Blantyre-rural).* Even though they knew condoms could be used to prevent pregnancy, they engaged in unprotected sex. Unprotected sex occurred because they lacked condom negotiating skills or considered unprotected sex a way of impressing their partner or making them happy. Sex, to them, was a symbol of love, even if they were fully aware of its potential risks. Their decision to engage in sex was impulsive, done without fully considering its consequences.

#### Inaccessibility of contraceptives and misconceptions

Previous studies [27, 28] have shown that access to and use of contraceptives remain limited among adolescents in Malawi, consequently, the prevalence of early and unintended pregnancy is high. Although awareness of contraceptives has significantly increased among adolescents, early unintended pregnancy has not significantly declined[29]. Our analysis reveals that most adolescents were aware of contraceptives and could name different types of contraceptives, including condoms, injections, pills, and implants. However, their awareness of contraceptives was not equivalent to having accurate knowledge of contraceptive methods, nor did it translate to their use. Recalling how she got pregnant, one 17-year-old girl explained: *“Yes, I knew about it [contraceptives] but did not have access to it.” (Participant 3, Female, 17 years, Blantyre urban).* Like many girls, she knew some pills could be used to prevent unwanted pregnancy, but she did not know how to access them. There is a community belief that young girls who had never given birth should not use hormonal contraceptives. Using hormonal pills before childbearing is believed to be the cause of infertility and young people without a child are discouraged from using them. One girl recounted:

“...at that time, I was not using any contraceptives because I had no child then and people always discourage young girls who have never given birth before to use contraceptives because it could affect the uterus so I was not using any contraceptives then.” (Participant 6, Female, 18 years, Blantyre rural).

This girl’s narrative illustrates how young girls are disadvantaged because they are excluded from using contraceptives like implants, intrauterine devices (IUDs), pills, and emergency contraceptives. Their only option is male condoms, whose use depends on their partners’ willingness and girls’ ability to persuade their partners to use them. Although some used condoms in their relationship they did not do so consistently, as recalled by one participant who became pregnant at 16 years: “*Yes, at the beginning when we were not faithful to each other we were using them. But sometimes we would not have them (Chishango [condom]) as a result we would just have the sex without any protection.” (Participant (4), Female, 19 years, Blantyre rural).* Inconsistent use of condoms is due to trust and unavailability.

#### School dropouts

When we asked what her reason was for becoming pregnant, one 17-year-old girl said, *“The big problem was that I stopped schooling.” (Participant (8), Female, 17 years, Blantyre rural).* When girls are out of school for extended periods, they become idle and fill up this empty time by entering relationships, often to pass time or meet their pressing needs. Previous studies have shown that keeping girls in school is protective against adolescent pregnancy [10, 30]. School takes significant time away from adolescents, limiting opportunities to meet up for sex. However, still, a significant number of girls are getting pregnant while in school. Girls drop out of school due to several reasons, including lack of school fees and materials, loss of interest in schooling, and COVID-19. Girls were sent away from school due to the inability of their parents to pay their school fees. It becomes difficult for adolescents to remain in school when they lack school fees and materials (e.g., uniforms) to support their continual education. When they are frequently sent away from school, they become demotivated and drop out of school:

The COVID-19 pandemic was noted to have further contributed to adolescent pregnancy with several adolescents reporting that they became pregnant during the COVID-19 school closures in 2020. Loss of learning hours means adolescents have idle time spent on dating and meeting up with partners. A few girls, however, dropped out of school because they thought it would allow them time to meet their day-to-day needs. They considered dating men as one means of meeting their needs as illustrated in the responses of one parenting adolescent girls:

“The big problem was that I stopped schooling. I stopped school and I thought this way I would get my needs to have a man aside me.” (Participant (8), Female, 17 years, Blantyre rural)

She dropped out of school to make room for a man in her life and having this man led to sexual intercourse and early pregnancy.

#### Sexual abuse

Early and unintended pregnancy may also result from sexual abuse, even though incidents often go unreported. Girls are abused by their partners and older men. Because they rarely report or seek care, they become vulnerable to unwanted pregnancy. One parenting boy response to the questions on reasons for becoming a father so early illustrates how sexual violence happens in relationships: *“I can say that she was both aware* [of contraceptives] *and also* [at the same time] *she was not aware, because I had forced her to have sex that was unprotected.” (Participant (3), Male, 19 years, Blantyre rural).*

The community often judge girls who become pregnant outside culturally approved unions and often label them as promiscuous or lacking self-control. Such judgmental attitude towards pregnant adolescents often ignores the fact that some of these pregnancies are due to sexual violence. One victim of sexual abuse in school recounted her experience:

“What happened is when I was still in school, my teacher used to give me money to spend so I would take it. At times he would send someone to come fetch me while I was in class. When the student came, she would tell me that ‘the teacher says you should go meet him’ and I would go. He would give me money and then start touching/caressing my breasts and then he would tell me to go. After that, I would go back to class. He would come anytime he pleased to my class and tell me to go to his class and I would go, he would give me money and then touch my breasts and it continued like that. During the school holiday when we were on break, he told me that we should meet at ADMARC and I went. On holiday, he sent a kid to come get me and the kid did that. He told me that I just gotten paid so let’s go on the road and I agreed. He booked a room and we slept there until morning. In the morning he gave me 4000 Kwacha to get a motorbike transport so that I can get back home. I took off and got home. That is the day I got pregnant.” (Participant (13), Female, 16 years, Blantyre rural).

She was in standard eight when this happened. He used condoms the first time they had sex. She recalled their first sexual encounter as coercion, with her refusing and him pressing her and running after her. She later succumbed to the pressure and pleaded with him to use condoms to avoid contracting HIV. On the second encounter leading to her pregnancy, she accepted to have sex unprotected in a hotel he took her after he had assured her that he was HIV-free. When she became pregnant, he refused to take responsibility for the pregnancy and had since abandoned her. Attempts to get him to take responsibility were unsuccessful given he had absconded the school. She narrated that she was unable to a health passport card to start antenatal health care at the clinic. She mostly depended on a friend to feed her and purchase the card. Following the birth of her child, she dropped out of school completely and was no longer living with her parents.

Another girl also shared about sexual abuse leading to pregnancy. In her case the abuser was a relative—her cousin; and once she became pregnant and the abuse was reported to the community leader but no action was taken: “*He just said that since we are related there was nothing he could say or do about it” (Participant 4, Female, 19 years, Blantyre rural).* Although the girl’s mother tried to report the abuse, it was to no avail: *“My mother said we should go to the police, but my grandmother refused…I do not know why but maybe because we [the abuser and my family] are related.*” It seems the community attitude toward relatives, even in abusive circumstances, is to protect family relations.

#### Poverty

Besides the lack of contraceptives, adolescent pregnancy was also fueled by poverty. A few girls recounted how they thought they could meet their needs through dating. Needs like clothes, food, and soap, among others, are not adequately provided by their parents because they are poor. When these girls depend on men to meet their needs, they are unable to decline unwanted sex or insist on condom use. The lack power and confidence to direct the course of the relationship. Such exchange of sex for money and material gifts disempowers girls and increases their vulnerability to early and unintended pregnancy. While the literature [31] has shown that poverty is not the only driver of transactional sex since other factors such as gendered power structures are influencers [31], girls interviewed in this study blamed poverty for their involvement in such relationships and ultimately for their pregnancy as recounted by an adolescent living with disability: “*The main reason* [for having unprotected sex] *was to get money and be able to buy myself clothes and other necessities.” (Participant (14), Female, 18 years, Blantyre rural).*

### Parental perceptions about the drivers of adolescent pregnancy

Parents had contrasting views on reasons for adolescent pregnancy. For example, while many adolescents attributed their pregnancy to being naively in love, lack of contraceptives and sexual violence, most parents felt that their daughter’s failure to adhere to advice to remain abstinent, peer influence, inadequate parental supervision, COVID-19 related school closure, and poverty were among the main reasons for adolescent pregnancy. Narratives of parents can broadly be grouped into two groups: self-blame and adolescents’ culpability.

Those who took responsibility blamed their failure to adequately provide for their adolescents’ and their failure to properly supervise them for the pregnancy. One mother recounted: *“mostly I blame myself because I was not able to give her everything that she could desire so I blame myself.*” *(Parent (7) Kapeni, Blantyre rural)*. Another parent blamed herself because she did not support her daughter sufficiently with finances for school fees, which led to her daughter dropping out of school. She further stated that it was during this dropout period that her daughter started engaging in sexual relations, and this led to her becoming pregnant. She shared her views on why her daughter got pregnant: *“Yes, so the interest in school was there but she could be sent home because of the school funds, and I could her send them back to school telling her that I will pay when I find money and she could be sent home again and again and that demotivated the child. And because of the spirit of just staying at home since she is being sent home from school, she just got used staying at home.” (Parent (7)*, *Makata, Blantyre urban).*

One guardian blamed her own absence and lack of supervision for her daughter’s pregnancy. When asked what the reason for her daughter’s pregnancy was, she responded by stating: *“I think I am the major cause for this because had I been here, I do not think it would have gotten this far. But all this happened because I was not here.” (Parent (2), Kapeni, Blantyre rural).*

However, most parents placed the blame entirely on their adolescents. They recounted how failure to follow parents’ advice around being abstinent, adolescents’ stubbornness and peer influence led to their daughters’ pregnancy. Many parents recounted how they tried in vain to stop their daughters from dating and warned them about the negative health and socio-economic effects of adolescent pregnancies. They spoke specifically about the risk of maternal deaths, the toll of pregnancy on physical health, the resources required to care for children, and the risk of sexually transmitted infections. Some parents resorted to corporal punishment, spiritual help, and the police as recounted by one mother: *“stubbornness, because we tried every way like going to the police, flogging her, advising her, and even praying for her but to no avail.” (Parent, Mbayani, Blantyre Urban).*

Some parents narrated how negative peer influence led to their daughters’ pregnancies. These parents believed that their daughters are well behaved at home but acted differently when in school and with friends. The blame their daughters’ failure to follow advice on negative influence of friends. The response of one parent illustrates this point: *“Herself, as you already know children go to school, they meet friends there. We don’t know what they talk about. We don’t know what they do. We only know they were doing unnecessary things when the damages are already done.” (Parent, Katundu, Blantyre Rural).*

A few parents, however, blamed school closure. These parents believed schools are protective against adolescent pregnancy because going to school limits girls’ free time for relationships with boys. One parent recounted: *“Closure of schools because due to closure of schools, lot of girls in my community have ended up in the same problem. You will meet so many similar problems in my community. As parents we believe that that was the main challenge because when children are in school, they might be attending part time classes hence going home late so they can’t do stupid things while in school.” (Parent, Lundu, Blantyre Rural).*

### Discussion of findings

The findings presented in this study provide unique insight into circumstances surrounding adolescent pregnancy in the Malawian context. In most cases, adolescent pregnancies are unplanned, occurring outside of wedlock, and while many are still in school [32]. Sex among adolescents occurs with little consideration of its consequences. Experiencing a sweep of new emotional impulses in relationships for the first time was more vivid for an adolescent than the realities of the outcomes of sexual behavior. As Jewell et al., [33] puts it “Sexuality carries its rationalities, which do not necessarily prioritize safe sexual behavior.” Emotional attachments also dictated the use of contraceptives or not; with adolescents who wanted to prove their trustworthiness and faithfulness engaging in unprotected sex and exposing themselves to unintended pregnancies.

Our findings on how poverty, lack of contraceptives, and sexual violence contribute to adolescent pregnancy are consistent with previous literature [10–12]. There is evidence that sexual violence and poverty are associated with a higher probability of adolescent pregnancy. As found in our study, girls living in poverty are tempted to meet their needs through dating. In such transactional relationships, girls lack the power to make decisions, communicate or negotiate condom use. Such relationships also expose them to sexual violence as money is used to entice poor girls into unwanted sexual relationships. Girls also lack the agency to use contraceptives and are not savvy on various methods of preventing unwanted pregnancy.

Although access to modern contraception has significantly improved over the past two decades, many adolescents remain vulnerable to early and unintended pregnancy. Also, the vulnerability of adolescents to unintended pregnancy is at odds with the government’s commitment to guaranteeing women and girls the right to reproductive health information as articulated in several forward-looking international and regional human rights frameworks like CEDAW and Maputo Protocol. International conference documents such as the ICPD Program of Action further reaffirm adolescents' rights to information and reproductive health education. In Malawi, policy documents provide for adolescents' access to sexual and reproductive health information and services in fulfilling international and regional commitments. However, a huge proportion of girls continue to get pregnant showing they lack access to sexual and reproductive health information, particularly accurate information on contraceptives.

We also found that sexual coercion is common in adolescent dating relationships, leading to unintended pregnancy. A previous study shows that adolescent girls leave decision-making, communication, and negotiations within sexual behavior to their male counterparts, which is a disposition that is exacerbated by unequal power relations [34]. The notion of consent for sex was foreign among adolescent girls thus they leave all decision-making concerning sex to their partners. However, in retrospect, many adolescents admitted that in doing so, they acted ‘naïvely’. When girls found themselves in secluded places with boys, they were unable to reject sex even though they were unwilling. Some were manipulated by older men into having unprotected sex.

While society blames girls for being sexually abused and often discredits their account of the incident, the implications are victims of sexual abuse rarely come forward or seek care, leading to unintended pregnancy. In addition, abortion is legally and morally prohibited, living girls with no option other than to carry such pregnancies to term and with significant mental health implications. A recent school survey in Malawi reveals how girls are exposed to sexual violence in school[35]. School environment can be unsafe for girls, with older men preying on students and enticing them with money and material girls.

The pathway through which transactional and coerced sex result in unintended pregnancy is through lack of contraceptive knowledge and use. Their ability to use condoms is hindered by adolescent girls’ inability to negotiate condom use and their limited access to condoms. Social norms around contraceptive use by adolescent girls and fears about the side effects of contraceptives also limit contraceptive options for girls. These findings concur with previous research by Bhatt et al*.* [36]*,* which shows that fears of side effects, such as fear of infertility by the community at large, leave an impression on, and thus influence girls’ early uptake of contraceptives. The findings suggest that community attitudes about sex education influence adolescents’ sexual behavior. To be successful, the approach to sex education must therefore go beyond the classroom setting and instead engage the attitudes of parents, community leaders, and men and women within the day-to-day community settings in which adolescents experience everyday life.

Our findings illustrate how parents generally hold contrasting views relative to adolescents. Parents, in general, did not consider the role of sexual violence and lack of contraceptive knowledge as reasons for their daughters' unintended pregnancies. Instead, they blamed adolescents for failing to follow their advice on abstinence, assuming girls in all cases willingly engaged in sex. Results suggest that parents focused on counseling their daughters on abstinence but did not educate them on contraceptive use. The implications of this are that adolescents are likely sourcing information about contraceptives outside their family home, and if the information they seek is lacking and they are facing challenges in heeding to parents’ advice, this can lead to unintended pregnancies.

Previous studies have found early and unintended pregnancy as one of the consequences of COVID-19-related school closure [37–40]. Keeping girls in school is generally considered to be an important intervention for preventing early and unintended pregnancy[41]. As such, the findings on the role of school dropout and COVID-19 school closure in adolescent pregnancy are expected. This finding highlights the need for continual investment and attention toward girls’ education as a strategy to reduce early and unintended adolescent pregnancies.

Generally, what is needed to help adolescents avoid unplanned pregnancies is to consider and acknowledge the emotional rationalities of most adolescents. Based on our findings, preventing unplanned pregnancies will require altering community attitudes about young people’s use of contraceptives and engaging parents, the education sector, civil society organizations, and community and religious leaders to develop comprehensive sexuality education programs to empower in and out-of-school adolescents.

### Study limitations

This study has both strengths and limitations. Our study provided in-depth analyses of the drivers of unintended pregnancies among those who have experienced early—and often unintended—pregnancies. It adds a relevant dimension reflecting the challenges of the current times, given that most of the adolescents became pregnant during the COVID-19 school closure period. However, our study is limited given that the views of parents in this study included only those of mothers. Future studies should incorporate the perspectives of fathers on drivers of unintended pregnancies.

## Conclusions

The reasons for adolescent pregnancy are complex and interconnected. Structural factors such as poverty and sexual violence contribute significantly to adolescent pregnancy in Malawi, suggesting that multilevel interventions are needed to address this problem. Our findings also contribute to the evidence of the continued vulnerability of girls to unintended pregnancy and document how divergent views between girls and their parents may contribute to the lack of progress in reducing adolescent childbearing.

## Data Availability

Data will be made available on reasonable request to the corresponding author.

## Abbreviations

CEDAW: Convention on the Elimination of all Forms of Discrimination Against Women
EA: Enumeration area
ICPD: International Conference on Population and Development
IUD: Intrauterine device
UNIMAREC: University of Malawi Research Ethics Committee
